# The impact of mammography screening programmes on incidence of advanced breast cancer in Europe: a literature review

**DOI:** 10.1186/s12885-018-4666-1

**Published:** 2018-09-03

**Authors:** M. J. M. Broeders, P. Allgood, S. W. Duffy, S. Hofvind, I. D. Nagtegaal, E. Paci, S. M. Moss, L. Bucchi

**Affiliations:** 1 Radboud Institute for Health Sciences, Radboud university medical center, PO Box 9101, 6500 HB Nijmegen, The Netherlands; 2 Dutch Expert Centre for Screening, Nijmegen, The Netherlands; 3 Centre for Cancer Prevention, Wolfson Institute of Preventive Medicine, Queen Mary University of London, London, UK; 4 Cancer Registry of Norway, Oslo, Norway; 5 Department of Pathology, Radboud University Medical Center, Nijmegen, The Netherlands; 6 Retired, Clinical and Descriptive Epidemiology Unit, Cancer Research and Prevention Institute (ISPO), Florence, Italy; 7 Romagna Cancer Registry, Romagna Cancer Institute (Istituto Scientifico Romagnolo per lo Studio e la Cura dei Tumori, IRST, IRCCS), Meldola, Forli, Italy

**Keywords:** Breast cancer, Mammography, Screening, Advanced stage, Review

## Abstract

**Background:**

Observational studies have reported conflicting results on the impact of mammography service screening programmes on the advanced breast cancer rate (ABCR), a correlation that was firmly established in randomized controlled trials. We reviewed and summarized studies of the effect of service screening programmes in the European Union on ABCR and discussed their limitations.

**Methods:**

The PubMed database was searched for English language studies published between 01-01-2000 and 01–06-2018. After inspection of titles and abstracts, 220 of the 8644 potentially eligible papers were considered relevant. Their abstracts were reviewed by groups of two authors using predefined criteria. Fifty studies were selected for full paper review, and 22 of these were eligible. A theoretical framework for their review was developed. Review was performed using a ten-point checklist of the methodological caveats in the analysis of studies of ABCR and a standardised assessment form designed to extract quantitative and qualitative information.

**Results:**

Most of the evaluable studies support a reduction in ABCR following the introduction of screening. However, all studies were challenged by issues of design and analysis which could at least potentially cause bias, and showed considerable variation in the estimated effect. Problems were observed in duration of follow-up time, availability of reliable reference ABCR, definition of advanced stage, temporal variation in the proportion of unknown-stage cancers, and statistical approach.

**Conclusions:**

We conclude that much of the current controversy on the impact of service screening programmes on ABCR is due to observational data that were gathered and/or analysed with methodological approaches which could not capture stage effects in full. Future research on this important early indicator of screening effectiveness should focus on establishing consensus in the correct methodology.

**Electronic supplementary material:**

The online version of this article (10.1186/s12885-018-4666-1) contains supplementary material, which is available to authorized users.

## Background

A long follow-up is required to assess the impact of mammography screening programmes on breast cancer mortality. The advanced breast cancer incidence rate (hereafter briefly referred to as advanced breast cancer rate, ABCR) can potentially be used as an earlier indicator of the effectiveness of a screening programme. Moreover, since tumour stage at diagnosis is independent of treatment, except for neoadjuvant therapy, analysis of trends in ABCR allows the effects of early detection to be disentangled from those of improvements in treatment [1]. The correlation between reductions in breast cancer mortality and ABCR has been firmly established on the basis of screening trials [2]. In a pooled analysis of data from eight trials, the decrease in the risk of advanced breast cancer and the decrease in the risk of dying from the disease were approximately proportional [1, 3]. It is clear that screening is associated with a reduction in the proportion of advanced stage cancers [4]. However, observational studies published over the last 15–20 years have yielded conflicting results on the association between the introduction of population-based service screening programmes and changes in ABCR, i.e. the absolute incidence of advanced stage disease [3, 5]. Nevertheless, the evaluation of the change in the incidence of advanced breast cancer cases is relevant in service screening outcome research. An apparent lack of this change has been considered by some as evidence of the lack of mammography screening programmes’ effectiveness [5–8].

The objectives of the current study were (a) to review studies of the effect of mammography screening programmes in Europe on ABCR, and (b) to summarize their limitations and the extent to which they contribute to the evidence on screening effectiveness.

## Methods

### Search strategy and selection criteria

A systematic search of PubMed with the search terms ‘cancer stage’, ‘screening’, ‘breast cancer’, ‘incidence’, and ‘mammography’ was performed to identify papers published from January 2000 until May 2013 (details in Appendix) and later updated to June 2018. Only papers in English evaluating European programmes were reviewed. The search strategy was built using 7 key papers [9–15].

Abstracts from the papers identified were reviewed by two from a group of four reviewers (MB, PA, SM, LB) and papers for full review were selected using the following general criteria: (a) the study represented original data and estimated the impact of a current regional or national population-based screening programme in Europe; (b) definition of advanced disease was based on breast cancer size, nodal status and/or stage at diagnosis of breast cancer; (c) the analysis included at least some of the age groups between 50 and 69; (d) the study used an observational research design comparing rates or proportions of advanced stage cancers; and (e) an uninvited and/or unscreened control population was available. This included the pre-screening years for the population targeted for screening in the study. Comparisons only of attenders vs non-attenders were not included. We focused the review on European programmes to add evidence on advanced breast cancer to the European balance sheet of benefits and harms as an outcome to the work of the Euroscreen reviews of observational mortality studies [16].

### Definition of advanced breast cancer

Tumour staging criteria vary across studies and even studies using the UICC TNM classification [17] show little agreement in their definition of advanced breast cancer. Theoretically, the benefit of screening is limited to screen-detected cases, either earlier within the same stage or at an earlier stage. However, using stage in itself has a disadvantage due to the stage migration bias caused by the introduction of sentinel lymph node dissection [18] and by changes in coding and classification practices [19]. In this respect, using only the pT information as a proxy for the diameter of the lesion is the most direct link to radiological detection and less influenced by trends in missing data and changes in coding and classification practices, even though it cannot show within-stage shifts in diameter. It is therefore the least biased option to define advanced breast cancer detection. Tumour size (measured in mm), even though put forward by some authors as an indicator of diagnostic anticipation [20], has never been confirmed as such and is often inaccurate since pathologists tend to round to the nearest multiple of five (terminal digit preference bias) [21].

### Theoretical framework and checklist

We designed an assessment form to extract detailed quantitative and qualitative information, the study design, completeness of information and results from the selected papers in a standardized fashion.

**Fig. 1:**
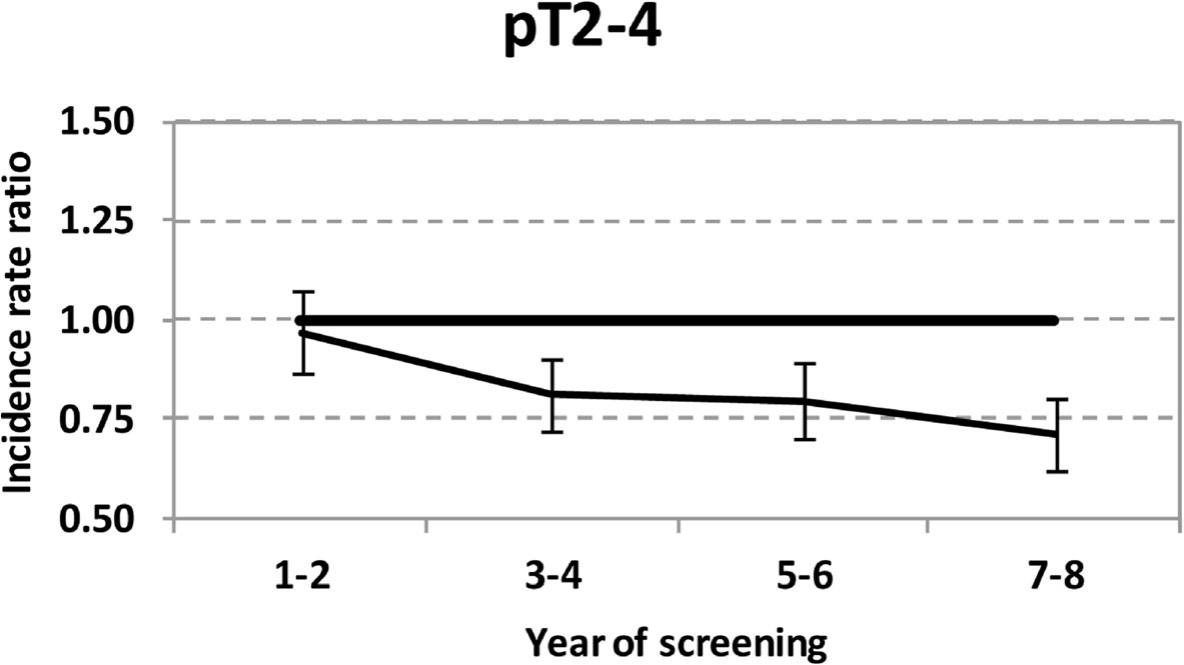
Expected effect of mammography service screening on the occurrence of advanced breast cancer, illustrated by Fig. 2, right panel, from Foca et al. [15]. Ratios with 95% confidence intervals are illustrated between the observed and expected age-standardised incidence rates of breast cancer per 100,000 women according to a 2-year screening period (ages 55 to 74 years). pT indicates pathologic tumour classification

The expected effect of mammography service screening programmes on ABCR is best understood looking at the randomized controlled trials (RCTs) as a reference, as previously described [1–3]. Based on the RCTs, the ABCR in the population invited to screening, usually from age 50, is expected to remain stable or slightly increase when the programme starts. The increasing incidence, in comparison with the prescreening incidence rate, is due to the intra-stage shift. This means that screening will detect advanced cancer cases earlier, but still within the same stage as in the absence of screening. After the prevalence screening, assuming a 100% sensitivity, the advanced cancer cases will be diagnosed as interval cancers, if fast growing, or are expected to be detected earlier at subsequent rounds. For this reason, the expectation is a reduction of the ABCR 2–3 years after the start (Fig. 1). The advanced cancer cases that are detected earlier through screening than they would have been in the situation without screening are the ones which should benefit. The ABCR should thus decrease from the time of prevalent screening (time 0) to a lower level than the expected, reaching a plateau after a few years, because screening will move diagnoses of breast cancer cases forward in time as long as the programme continues. If screening stops, e.g. at 65 or 69 years in most European screening programmes, the ABCR is expected to increase again, rising after some years to the prescreening level (age-specific) .

In order to discern this pattern of occurrence, the ABCR with or without screening will be best observed in a study where individual women are followed over time, and an unconfounded comparison of screening with non-screening incidence is available. In order to assess the extent to which studies achieve or approximate this ideal situation, we developed a ten-point checklist of the main methodological issues with which such studies of ABCR have to contend, logically derived from the above described theoretical framework (Table 1). The checklist is based on epidemiological principles of observational studies as applied to screening [22] and previous research experience, including knowledge of the relevant literature from outside of Europe [6, 7, 23–26] and findings of the Euroscreen reviews of observational mortality studies (trend studies, incidence-based mortality studies, and case-control studies) [27, 28]. The methodological issues identified using the ten-point checklist, their definitions, and their consequences on design, likely accuracy, and results of studies are presented in Table 1. This in turn highlights the main potential departures of studies from the ideal design of a study of the temporal association between mammography screening programmes and incidence of advanced stage breast cancer, and indicates the major issues of interpretation of the results.

**Table 1:** Ten-point checklist of main methodological problems affecting studies of the effect of mammography screening programmes on the incidence of advanced breast cancer

Point #	Issue	Problem	Consequence	Potentially affected studies (reference number)
1	Follow-up time	The time window available to observe a decrease (if any) in ABCR is narrow and closes rapidly. In the Two-County trial, ABCR in the study group began to decrease 4 years after randomization and stabilized at a lower level on the 8th year [2].	The ABCR is expected to increase with the prevalence screening, it may fall in the years immediately following the prevalence screen, and will likely be stable at the end of screening in a cohort of women. In trend and dynamic population analysis, in the absence of an individual time zero (time at entry), the effect is confounded and the effect of screening on ABCR is underestimated. This is particularly applicable to estimates of annual percent change.	[8, 12, 13, 19, 34, 37, 41]
2	Exposure time	The target population is a dynamic one (but the same holds true for cohort studies). Because there is a latency for the effect of screening on ABCR to take place, at any point in time there are women (i.e., new quinquagenarians, new immigrants, and late attendees) with insufficient exposure time.	The effect of screening on ABCR is underestimated, due to a disproportionate influence of prevalence screens.	All studies
3	Pace of implementation	Public health screening programmes are implemented gradually, in a markedly stepwise fashion, since large populations are divided in distinct administrative units each targeted by an independent local plan of action.	The effect of screening on ABCR is diluted. Until implementation is completed, there are women who are diagnosed with breast cancer before being invited, and who greatly contribute to ABCR.	[8, 14, 15, 19, 29, 30, 32, 33, 36–39, 44]
4	Prevalence effect	The prevalence screen may be associated with a transient increase in ABCR [13].	During a stepwise implementation of the programme, when the time elapsed from the start is theoretically sufficient to see a decrease in ABCR, this is counteracted by an opposite effect due to newly enrolled women – especially if invitations increase over time.	[8, 14, 15, 19, 29, 30, 32, 33, 36–39, 44]
5	Reference incidence (i)	The reference (or underlying) incidence rate, with which to compare the rate observed after the introduction of screening, is not known with precision [49].	The rate can be estimated using the rate observed in the last few years before screening, assuming its stability over time, or by linear extrapolation of a pre-existing trend. The second approach is arguably preferable, but both are dependent on underlying assumptions about trends or absence of trends in incidence, and results can vary depending on these assumptions.	All studies
6	Reference incidence (ii)	Whatever incidence rate is being used as a reference, its validity decreases with increasing number of years of observation due to uncontrollable changes (or in the pace of such changes) in the underlying risk of breast cancer.	Assessing the long-term effect of screening on ABCR is subject to considerable uncertainty and there is potential for inaccuracy in either direction (overestimation or underestimation of effect).	[8, 12, 13, 19, 34, 37, 41]
7	Definition of advanced cancer	There is no agreed definition of advanced breast cancer [50], even though there is general agreement that large or metastatic cancers are ‘late stage’.	The definition is chosen based on differing criteria. The pT information alone, which is the most available one, is direct and relatively unaffected by biases due to confounding. Conversely, multiple-stage data are more meaningful, since the effect of screening may differ across different categories of advanced cancers.	All studies
8	Stage migration	The introduction of sentinel lymph node biopsy between mid-1990s and mid-2000s caused a substantial increase in the registered incidence of node-positive breast cancer (stage migration bias) [18].	The use of pN staging is problematic in studies of trends in ABCR over the last two decades, since changes in the risk of node-positive cancer cannot be adjusted for stage migration. The increase in node-positive disease is likely to be population-specific and will depend on the rate of change of local surgical policy. However, reductions in node-positive disease as a results of screening are likely to be underestimated rather than overestimated due to the stage migration.	[12–14, 19, 29–43]
9	Missing data on tumour stage	Whatever staging system is being used, the introduction of a screening programme tends to bring an improved quality of breast cancer registration, with a sharp decrease in the proportion of unknown-stage cancers.	Because more cases are increasingly placed in all known-stage categories, an apparent increase in all stage-specific rates occurs – including ABCR.	[8, 15, 30, 32, 33, 38, 39]
10	Statistical approach	The statistical approach is not standardised, and includes the provision of purely descriptive information and the use of methods which are difficult to interpret, such as joinpoint analysis.	Descriptive information does not allow evaluation of the magnitude and significance of observed changes in ABCR. Methods like the joinpoint analysis are useful for assessing the points in time when ABCR begins to decrease and when it stabilizes, but may be misleading when used to assess the significance of the trend. Also, the important issue is arguably what happened to ABCR following the screening rather than at what point a change occurred in the direction of a trend, which is affected by both confounding and analytic assumptions.	[8, 12, 13, 19, 29, 35, 40–43]

The checklist items included: 4 complications related to the timescale of screening introduction, periods of exposure and observation, and transient prevalence screen effects; 3 to endpoint definition, stage migration and completeness of stage data; 1 to difficulties of formal inference; and 2 to the inevitable problem of incomplete information on what the incidence of breast cancer overall and of advanced disease would have been in the absence of the screening programme.

### Presentation of results

Due to the heterogeneity in methodology and endpoints used in the studies, no attempt was made to produce a pooled estimate of the effect of screening on ABCR. Instead, we reported details of methods and results of each study individually in Additional file 1: Table S1. We looked for data on screening coverage and attendance rates from other sources as well, if the selected study did not provide that information.

## Results

### Selection of studies

**Fig. 2:**
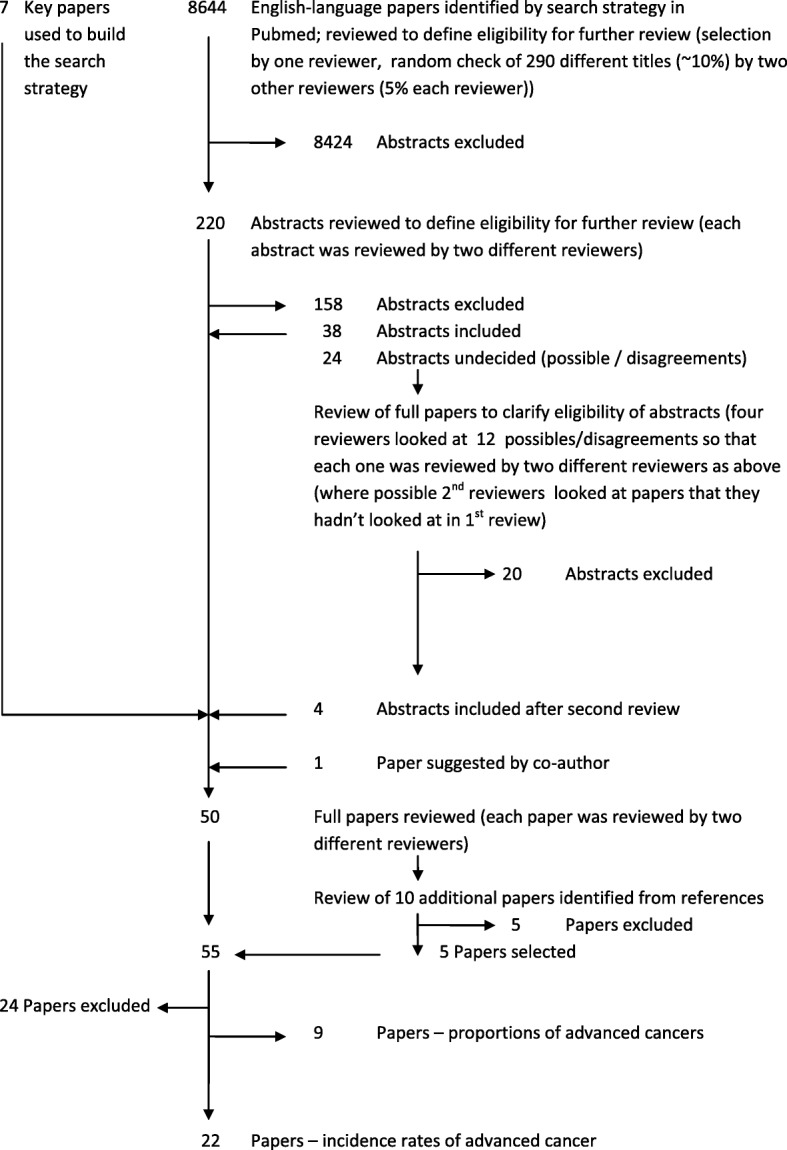
Flowchart of search strategy and selection of papers

The search strategy identified 8644 English-language papers of which 220 were considered relevant based on title and abstract (Fig. 2), including both studies of incidence rates and those of proportions of advanced cancers.

Based on the selection criteria, 38 studies were included, and a further 24 were identified as possible inclusions. For the latter group, full papers were assessed by two different reviewers, with arbitration by a third (SD) where necessary, which resulted in the inclusion of 4 studies. In addition, the abstract of one paper suggested by a co-author was assessed and included for review. In total, after adding the 7 key papers, 50 studies were included for full paper review by the two reviewers who had not assessed the abstracts. We also manually searched the reference lists of these papers and identified 10 references that fulfilled the inclusion criteria but had not been identified by the search strategy. Review of the full papers for these references resulted in the inclusion of an additional 5 studies. Differences between reviews were resolved through consensus by all four reviewers. Of the 60 full paper reviews in total, 22 studies were found eligible for inclusion in a comparison of incidence rates as the outcome measure [8, 12–15, 19, 29–44]. A further 9 studies were comparisons of proportions of advanced cancers and not included in the current review. Of the 29 papers excluded, 21 lacked a suitable control group, 3 were not related to population-based screening and 5 were excluded for other reasons (no data for age group 50–69 (*n* = 2), no tumour stage data (*n* = 1), not European Union (*n* = 1), and no original data (*n* = 1)).

### Study generalities

These are shown in Additional file 1: Table S1. The 22 eligible studies were from Norway (*n* = 5), Italy (*n *= 5), the Netherlands (*n* = 4), Denmark (*n* = 2), Sweden, Finland, Germany, United Kingdom (UK), Ireland, and France. There were 9 nation-wide studies, four from Norway [19, 36, 38, 39], two from the Netherlands [14, 41], two from Denmark [8, 37], and one from Finland [34].

### Programme characteristics

In most studies, the target age range was 50–69 years [8, 14, 15, 19, 29, 30, 32, 35–41, 44] or wider [12, 31, 43]. The papers from Finland, the West Midlands region of the UK, and Ireland reported programmes aimed at women aged 50–59 years [34] and 50–64 years [13, 42]. The target age of the Swedish programme varied locally between 40 and 74 years [33]. The size of the target population, often not reported, was between 500,000 and 1,000,000 in the national Dutch study [14], in the Danish studies [8, 37] and in one Italian study [15], and exceeded 1,000,000 in the study from Sweden [33] and in a second study from Italy [32]. The screening interval was 24 months except in the West Midlands (36 months) [13]. The start of screening programmes ranged from the early/mid 1970s in Florence, Utrecht, and Nijmegen [14, 29] to 2005 in the Münster district (Germany) [40]. The time period of observation of breast cancer incidence was between the second half of 1980s and the first half of the current decade in most studies.

### Study design

The methods of analysis varied from the provision of purely descriptive information to the evaluation of the magnitude and statistical significance of observed changes in ABCR. We assigned the design of the studies that evaluated the magnitude of effect to four broad categories:

1. comparison of ABCR before and after the introduction of screening using different endpoints, i.e., annual percent change (APC), percent reduction in ABCR, absolute reduction in ABCR, incidence rate ratio (IRR), relative risk (RR), excess RR, slope value calculated from a log-linear Poisson regression model, and observed:expected ratio, or simply by juxtaposition of rates [8, 12, 15, 19, 29, 30, 32–40, 43, 44];
2. comparison of ABCR between each year after the introduction of screening and the prescreening years using the estimated annual percent change (EAPC) [14, 31];
3. calculation of the EAPC after the introduction of screening without information on prescreening years [13, 41]; and
4. comparison of ABCR in an invited population vs. a neighbouring uninvited one using the percent reduction in ABCR. This is the case for a single study [42], although the inclusion of neighbouring nonscreening areas is a secondary part of the design of other investigations [8, 36].

The statistical significance of observed changes, if any, was assessed in 17 studies [8, 13–15, 30–34, 36–41, 43, 44].

Some information on the trend (before and after the introduction of screening) for the frequency of unknown-stage cancer was provided by 11 studies [8, 12, 15, 19, 29, 30, 32, 33, 35, 38, 39]. The tumour staging criteria varied. Although 20 studies used the UICC TNM classification, there was little agreement in the definition of advanced breast cancer. In one study, incidence was presented for multiple stage categories but the advanced category (or categories) was not explicitly identified [29].

### Study results

A significantly favourable impact on ABCR was reported by nine studies. In the national Dutch study, ABCR [T2+ with lymph node (N+) and/or distant metastases (M1)] decreased by 12% [14]. In one regional Dutch study, the annual IRR varied between 0.86–0.82 (T2+ cancer) and 0.83–0.72 (N+ cancer) [31]. In the study from Sweden, RRs were 0.74 (tumour size > 2 cm), 0.89 (N+ cancer), and 0.84 (Stage II+ cancer) [33]. In the national Finnish study, the ABCR (non-localised cancer) decreased by 9% [34]. A significant impact on ABCR was observed in three studies from Italy. Paci et al. found a RR (Stage II+) of 0.72 [30]. The figure reported by Foca et al. for T2+ cancer was between 0.81–0.71 [15]. A secondary observation from a more recent Italian cohort study comparing attenders and non-attenders was a significant ratio of 0.83 between the observed number of T2+ cancers in a whole invited cohort and the expected number based on pre-screening rates [44]. In a large French study, the decrease was significant both for T2+ cancer and Stage II+ cancer [43]. In a local study from Germany, Simbrich et al. demonstrated significant decreases of varying magnitude in annual ABCR among women aged 50–69 years [40].

Two studies provided unclear results. A Danish study described a transient increase in incidence of cancers > 20 mm in size in early screening regions followed by a decline of N+ cancers in late screening regions [37]. The Italian study of Buiatti et al. was limited to ≤3 screening years for most of the participating subareas. After early significant increases in T2+ cancer rates in two of them, a moderate reduction was observed 4–6 years after the start of the programme in the area with longer follow-up [32].

Four nationwide Norwegian studies reported contradictory findings. Kalager et al. observed a significant IRR (Stage III+ cancer) of 0.76, but the same figure was found in the not-yet invited population before screening [36]. Also, the reduction was confirmed by a second study but in association with an increase for Stage II cancer [39]. Others reported the opposite, that is, a decrease for Stage II cancer and an increase for Stage III cancer [19]. Another study found significant increases both for Stage II and Stage III cancers and a decrease for Stage IV cancer alone [38]. None of these studies used individual data indicating whether women were diagnosed before or after they were invited to participate.

In addition to the abovementioned studies from France [43] and Germany [40], three investigations used the joinpoint analysis or the Poisson regression analysis. In the West Midlands (UK), the incidence of N+ cancer increased in the first years of screening and then returned to the baseline level but with a significant positive APC of 1.1 [13]. In Denmark, the negative APC in incidence of T2+ cancer was significant but the ratio between post-screening and pre-screening rate was not significantly different from the unity [8]. In another study from the Netherlands, a non-significant negative APC in Stage 2+ cancer rate was observed but the estimate included the whole of women aged 50 or older [41].

Four studies, in addition to one of the abovementioned Norwegian studies [19], presented no assessment of significance of observed changes in ABCR (if any). One Italian study reported a 8.7% decrease for N+ cancer [29]. In the fifth Norwegian study, ABCR (regional or distant cancer) rose before the introduction of screening, and fluctuated thereafter at levels that were generally above the last pre-screening level [35]. In a regional Dutch study, ABCR (Stage IIA+ cancer) was described to be stable before and after the introduction of screening [12]. In Ireland, ABCR (Stage 2+) in a region targeted by screening in 2000 fell by 20% in comparison with a region in which screening was implemented only seven years later [42].

### Method check

The right-hand column in Table 1 gives the results of the review of selected papers against the ten-point checklist.

The issue of follow-up time (#1) is related to the short time window after prevalence screening where a decrease in ABCR can be observed. Studies with a long time window, most notably seven studies [8, 12, 13, 19, 34, 37, 41] in which the time difference between the year of start of the screening programme and the last year of observation was ≥15 years, will not be able to show this decrease. This is particularly problematic when interpreting annual percent changes [13, 41]. If screening is working as anticipated, annual percent changes will be substantial in the first years of a programme, but will be small or absent after the programme has achieved widespread coverage as the new lower incidence will be roughly constant. The related problem of the effect of a dynamic population on exposure time (#2) applies to all studies. Foca et al. excluded women aged 50–54 years but not new immigrants and late attendees [15]. Anttila et al. provided separate data for women aged 50–54 years and 55 years or older [34].

The problem due to pace of implementation (#3) applies especially to the Swedish study [33], the Italian studies [15, 29, 30, 32, 44], the nationwide Norwegian studies [19, 36, 38, 39], the Danish studies [8, 37], and the nationwide Dutch study [14]. In fact, it is rare that a mammography service screening programme is started simultaneously throughout a large geographic area. In two of these studies, there was explicit adjustment of the analysis to address this issue. In the Swedish study, the first screening years in some counties were omitted from analysis because mammography coverage, or the level of exposure, was still low [33]. In addition, in this study, individual data on screening exposure was available for the nominal screening period. In the study of Foca et al. the years of observation were synchronised at the municipality level, and those municipalities where saturation was not reached within a short (arbitrary) time interval were not taken into consideration [15]. This proved to be a practical but powerful approach to account for gradual programme implementation. In other studies, at least some information was available for the reader to assess the potential size of the problem. The papers reporting the nationwide Dutch study and the Danish study drew the reader’s attention to this issue by presenting results for individual years and for regions implementing screening at different times [14, 37]. One of the Italian studies also had individual data on screening exposure during the nominal screening period [30].

The prevalence effect problem (#4) applies virtually to all studies with markedly stepwise implementation of the programme. Of the two problems concerning the reference incidence, the inevitable lack of a verifiable estimate of the underlying background incidence rate (#5) applies to all studies. Outside of a randomised trial, the estimation cannot be performed without assumptions regarding the likely incidence of breast cancer, and specifically late stage breast cancer, in the absence of screening. The problem of its decreasing validity over time (#6) applies especially to those studies, already mentioned above, in which the time interval between the last prescreening year and the last year of observation was ≥15 years [8, 12, 13, 19, 34, 37, 41]. However, again, presentation of data for individual years affords the reader a means of assessing the likely extent of underestimation [37].

Difficulties with the definition of advanced cancer (#7) apply to all studies, because all such definitions have pros and cons. Some used the pT information alone [8, 15, 44], others used multiple advanced stage definitions with separate results [13, 19, 29, 31, 33, 36–39, 43], or a single definition of advanced stage based on the TNM system [12, 14, 30, 32, 34, 35, 40–42].

Of the two problems concerning tumour stage information, the problem of stage migration (#8) applies to all studies except those where the definition of advanced cancer was exclusively based on pT information [8, 15, 44]. More than half of the studies did not take changes in the proportion of unknown stage information (#9) into consideration, providing no trend in missing tumour stage data [12–14, 31, 34, 36, 37, 40–44] or only very partial data [32]. A stable trend was reported by one of the Italian studies [29]. A percent decrease of incident breast cancers with missing stage information was observed in other two Italian studies [15, 30], in the Swedish study [33], in three Norwegian studies [35, 38, 39], and in a study from Denmark [8]. In two of these, the resulting bias was adjusted for in the design [15] and, respectively, in the analysis [33].

Finally, the problem of a lack of standardised statistical approach (#10) applies especially to those studies reporting purely descriptive data [29, 35, 42] or incidence curves without numerical data [12, 19] and those based on the joinpoint analysis [13, 41] and the Poisson regression analysis [8, 40, 43], the results of which are difficult to interpret.

## Discussion

The 22 studies included in this review showed considerable variation in results on the estimated effect of the introduction of population-based mammography screening programmes on the ABCR. Of note, there are four circumstantial indications that the overall effect of methodological issues resulted in an underestimation of the impact on ABCR: first, most biases have a conservative direction (#2, #3, #4, #8, and #9); second, most of the largest studies reported a significant decrease in ABCR [14, 15, 33, 44]; third, the decrease was more pronounced after some adjustments for design biases were made [15, 33]; and, fourth, taking the entire series of studies into consideration, nine of them found a significant, albeit varying, reduction in ABCR. They represent the majority of published studies once those affected by critical limitations are excluded. In our opinion, the report by Buiatti et al. [32], focusing the first 3 years of screening, and the four nationwide Norwegian studies [19, 36, 38, 39], with their conflicting and partly opposite findings, are difficult to interpret. Furthermore, the study by Larsen et al. demonstrated clearly that stage-specific incidence of breast cancer in Norway was influenced by changes in coding and classification practices, which makes it even more challenging to evaluate and compare stage-specific trends and stage migration of breast cancer by age and time [19].

Nonetheless, the conclusions of the available literature still warrant careful interpretation, because not all methodological concerns could be avoided. Also, while the direction of the potential biases can be predicted, it is difficult and sometimes impossible to estimate their magnitude. Some of the problems are unavoidable and apply to all studies (specifically #2, #5, #7), whereas others could potentially be addressed in the design phase. In any case, it would be arbitrary to rank their consequences in terms of relative impact on study results, which may also vary in relation to local contingencies. More realistically, we aimed at summarising the challenges in designing studies on ABCR in order to improve consistency in the reporting of results.

Ideally, the study population should be rapidly saturated by exposure to screening, and this should take less time than that needed for the expected effect on ABCR to become apparent. From this point of view population-based service screening programmes often cannot provide this ideal situation. The dynamic nature of the target populations, together with the phased introduction of most screening programmes and the fact that the prevalence screen will be associated with an increase in ABCR, will lead to an underestimate of the decrease in ABCR, as will the reduction in the proportion of unknown-stage tumours.

In addition, certain statistical analyses, such as the joinpoint analysis (#10), may generate false-negative results. Conversely, problems of estimation of underlying incidence in the absence of screening, and particular definitions of advanced stage (#5 and #7) may have been responsible for unpredictable effects in either direction. Many of the problems also arise from the reliability and validity of incidence data, in particular the unavailability of reliable reference incidence rates for advanced cancer, especially in a historical comparison period, together with the sharp decrease in the proportion of unknown-stage cancers following the introduction of screening. Stage migration bias, caused by the implementation of sentinel lymph node biopsy between the mid-1990s and mid-2000s [18, 19], will also have had an impact.

Furthermore, the inconsistency in the definition of advanced cancer gives rise to difficulties in interpreting the collected evidence. There is a possibility of a residual improvement within stage categories, but this is more difficult to demonstrate. The consistency between studies in the use of tumour diameter, stage and other parameters was limited. Another limitation in the classification of advanced cancers, especially in studies performed nowadays, is the variation among cancer registries (and within cancer registries over time) in what clinical and pathological data they collect. There is growing interest in the effect of screening, if any, on biological and molecular markers, but it will be some time before sufficient data are generated to answer this question. Incidentally, we believe that deficiencies in staffing, organisation, access, and funding of ongoing mammography service screening programmes warrant much greater consideration in the debate about their effectiveness.

From a scientific point of view, however, the most severe limitations of reviewed studies (#1 to #4) affected the study design. The main departures from the ideal design of a temporal correlation study were the following. First, as shown in the Swedish Two-County trial [2, 15], the time window available to observe an impact (if any) on ABCR closes rapidly. In populations where screening has been ongoing for a longer time [12, 13, 41], analysis should focus on establishing whether incidence of advanced disease is lower than before, not ‘still decreasing’. The misuse of the joinpoint analysis and of the Poisson regression analysis (#10) is itself related to the assumption that the downward incidence trend must continue indefinitely [13]. This cannot be the case, unless a substantial increase of mammography sensitivity occurs over time. Second, the 3-year latency of the effect of screening on ABCR means that, in the dynamic target population of a service screening programme, at any point in time, there is always a subset of women with an exposure time to screening that is too short to have an effect on the risk of advanced breast cancer. Third, and more important, service screening programmes in Europe were introduced very gradually. This inevitably caused the same dilution of effects as that historically described for cervical cancer screening in Denmark and Norway as compared with Finland and Sweden [34].

In fairness, most of the studies reviewed either attempted to control for possible problems by adjustment in statistical analysis or presented data in sufficient detail for the reader to judge the likely presence and direction of potential biases. There have been surprisingly few attempts, on the other hand, to adjust the design to minimise biases. The only previous literature review on ABCR following the introduction of mammography screening programmes did not take into consideration the limitations of published articles, except for the stage migration bias [5, 19]. The authors concluded that trends in advanced breast cancer incidence do not support a role for screening in the decrease in mortality. The present work demonstrates that the available literature cannot support such a conclusion, and indeed supports the opposite.

## Conclusions

In summary, all studies were challenged by multiple issues, although to a varying extent. The trend in most of evaluable results, even though inconsistent, does support a reduction in advanced breast cancer incidence following the introduction of mammography screening. In view of the impact on ABCR observed in RCTs [1], we conclude that much of the current controversy on mammography service screening programmes is due to observational data that were gathered and/or analysed with methodological approaches which could not capture stage effects in full [27, 28]. Notwithstanding this fact, changes in ABCR remain an important early indicator of effectiveness. Improving the knowledge of limitations in previous studies will help to establish consensus on the correct methodology. The development of more robust and empirically driven techniques should take into account both the practical implementation of cancer screening activities and the evaluation of their effects. This will enable a better fit of the design of studies on ABCR to the particular context of a mammography service screening programme.

### Additional file

Additional file 1: **Table S1.** Characteristics of the screening programmes, and design and results of studies of the impact of mammography screening on the incidence of advanced breast cancer. (*See the full text of the article for abbreviations*) [45–48]. (DOC 197 kb)

## Appendix

### Search strategy

(((((((((cancer stage[All Fields] OR cancer stages[All Fields] OR cancer staging[All Fields]))

OR (metastases)) OR (lymph nodes)) OR (lymphatic metastases)) OR (lymph nod*)) OR (tnm stage)) OR (tnm stag*))) AND (((((((early detection of breast cancer)) OR (population screen*)).

OR (mass screen*)) OR (mammogr*)) OR (cancer mass screening)) OR (mammography))

## Abbreviations

ABCR: Advanced breast cancer rate
APC: Annual percent change
CI: Confidence interval
EAPC: Estimated annual percent change
IRR: Incidence rate ratio
M1: Distant spread
N +: Node-positive
NA: Not applicable
NOS: Not otherwise specified
NR: Not reported
NS: Not significant
O:E: Observed:expected
pT: Pathologic tumour size category
RCT: Randomized controlled trial
RR: Relative risk
S: Significant
SOSSEG: Swedish Organised Service Screening Evaluation Group
T2 +: Tumour size > 2 cm
TNM: Tumour, Node, Metastasis
TX: Unknown tumour size
UICC: Union Internationale Contre le Cancer
UK: United Kingdom
W: Women
