# Supplementary material for: The impact of mammography screening programmes on incidence of advanced breast cancer in Europe: a literature review
Source: BMC Cancer. 2018 Sep 3;18:860. doi: 10.1186/s12885-018-4666-1 (PMC6122725; doi:10.1186/s12885-018-4666-1)
Supplement: Supplementary file 1 — Table S1. Characteristics of the screening programmes, and design and results of studies of the impact of mammography screening on the incidence of advanced breast cancer. (See the full text of the article for abbreviations) [45–48]. (DOC 197 kb) [file 12885_2018_4666_MOESM1_ESM.doc]

**Table S1. Characteristics of the screening programmes, and design and results of studies of the impact of mammography screening on the incidence of advanced breast cancer**

*See the full text of the article for abbreviations*

| **Study generalities** | | | |  |  |  |
| --- | --- | --- | --- | --- | --- | --- |
|  | First author | | Barchielli A | Paci E | Schouten LJ | Buiatti E |
|  | Year of publication | | 2001 | 2002 | 2002 | 2003 |
|  | Country | | Italy | Italy | The Netherlands | Italy |
|  | Regional area(s) | | Florence area (city of Florence and surrounding municipalities) | City of Florence | Limburg | 7 areas in central and northern Italy |
| **The screening programme** | | |  |  |  |  |
|  | Target age (years) | | 50-69 | 50-69 | 49-69 (49-75 since 1998 ) | 50-69 |
|  | Target population | | 164,000^b^ | 60,000 | NR | 1,033,000 |
|  | Screening interval (mos) | | 24 | 24 | 24 | 24 |
|  | Year of start | | Some municipalities, early 1970s; city of Florence, 1990; other municipalities, after 1992 | 1990 | 1990 | Locally varying between 1990-98 |
|  | Year of saturation^a^ | | After the end of the time period of observation (see Remarks) | 1993 | 1994 | After the end of the time period of observation |
|  | Response rate | | 60% | NR | First invitation, annually 25-82%; subsequent invitations, 77-85% | 65% [15] |
| **Study design and results** | | |  |  |  |  |
|  | | Time period of observation | 1985-94 | 1985-96 | 1987-99 | Prescreening years, locally varying between 1988-97; screening years, locally varying between 1990-99 |
|  |  | Design | Study of all-age incidence by stage in 1985-87, 1988-90, and 1991-94, with a focus on W aged 50-69 | Study comparing ABCR in 1990-96 (screening period) vs 1985-86 (prescreening period), and in invited W vs noninvited W | Study comparing ABCR in each year 1987-99 (screening years) vs 1987-90 (prescreening period) | Study comparing ABCR in the screening period vs the prescreening period, by area, in W aged 40-79 |
|  |  | Endpoint | % change in incidence rates, by stage, in 1991-94 vs 1985-87 | % and absolute reduction in ABCR, and invited:noninvited RR | IRR | IRR |
|  |  | Tumour staging | Tumour spread (local, regional, distant) | UICC TNM | UICC TNM | UICC TNM |
|  |  | Definition of advanced stage | None specified | Stage II+ | Distinct definitions: T2+, N+ | Tumour size >2 cm or N+ or Stage IV |
|  |  | Frequency of unknown stage cancer | Unknown tumour spread, stable incidence rate | Unknown stage: 1985-86, 14%; 1990-96, 7% | NR | Unknown stage, 6% with a significant reduction in the screening period in one area |
|  |  | Results | W aged 50-69: regional, -8.7% (significance, NR); distant, NR | % reduction in ABCR, -19; absolute reduction, -3.6 per 10,000; RR, 0.72 (95% CI, 0.59-0.87) | T2+: increase in 1991 (IRR, 1.22; 95% CI, 1.09-1.37), decrease in 1998 (0.86; 0.77-0.97) and 1999 (0.82; 0.73-0.92). N+: increase in 1991 (IRR, 1.28; 1.13-1.45), decrease in 1995 (0.83; 0.73-0.94) and 1999 (0.72; 0.63-0.81) | IRR by area, from 0.91 (*p* = 0.07) to 1.21 (*p* = 0.02) |
|  | Remarks | | By 1995, only part of municipalities of the Florence area were targeted by screening |  |  | The study was limited to ≤3 screening years for 5/7 areas. A moderate reduction in ABCR was observed 4-6 years after the start of the programme in one area |

**Table S1. CONTINUED**

| **Study generalities** | | | |  |  |  |
| --- | --- | --- | --- | --- | --- | --- |
|  | First author | | Fracheboud J | SOSSEG | Anttila A | Hofvind S |
|  | Year of publication | | 2004 | 2007 | 2008 | 2008 |
|  | Country | | The Netherlands | Sweden | Finland | Norway |
|  | Regional area(s) | | NA (nationwide study) | 13 counties | NA (nationwide study) | Rogaland, Akershus, Hordaland, and Oslo counties |
| **The screening programme** | | |  |  |  |  |
|  | Target age (years) | | 50-69 | Locally varying between 40-74 | Mainly 50-59 | 50-69 |
|  | Target population | | 813,000 in 1997 [45] | 4,403,000 person-years | NR | NR |
|  | Screening interval (mos) | | 24 | 24 in most counties | 24 | 24 |
|  | Year of start | | Utrecht and Nijmegen regions (“old” regions), mid-1970s; the 7 remaining regions (“new” regions), 1990-91 | Locally varying between 1988-96 | 1987 | Rogaland county, 1995; Akershus, Hordaland, and Oslo counties, 1996 |
|  | Year of saturation^a^ | | Approximately 1994 | NA | 1992 | NR |
|  | Response rate | | 78% [45] | 70-90% [46] | NR | First 10 years, national average 76% |
| **Study design and results** | | |  |  |  |  |
|  | | Time period of observation | 1989-97 | Prescreening epoch, locally varying between 1968-95; screening epoch, locally varying between 1988-2001 (see Remarks) | 1971-2002 | 1987-2004 |
|  |  | Design | Study of all-age incidence in each year 1990-97 vs 1989, by group of regions, with a focus on W aged 50-69 | Study comparing advanced cancer risk in the screening epoch vs the prescreening epoch (W aged 40-69) | Study comparing the observed ABCR in the years 1998-2002 with that expected based on extrapolation of rates from 1971 to 1986 in 5-year age groups between 50-69 | Study of ABCR in 1987-95 (prescreening period) and 1996-2004 (screening period) |
|  |  | Endpoint | EAPC in ABCR | RR of advanced cancer adjusted for the proportion with missing stage data and the increase in underlying incidence | Excess RR in % | ABCR |
|  |  | Tumour staging | UICC TNM | UICC TNM | Tumour spread (localised, non-localised). Non-localised (or regional/distant) spread mainly based on lymph node status | Tumour spread (local, regional, distant) |
|  |  | Definition of advanced stage | T2+ and (N+ and/or M1) | Distinct definitions: tumour size >2 cm, N+, Stage II+ | Non-localised spread | Regional or distant spread |
|  |  | Frequency of unknown stage cancer | TX, between 2.1% and 3.2% annually (no time trend data) | Tumour size unknown: prescreening epoch, median among counties 12%; screening epoch, 1%^b^ | Unknown stage (NOS), 9.4% (no time trend data) | Unknown regional spread, NR. Unknown distant spread: 1987-95, 2%; 1996-2004, 7%^b^ |
|  |  | Results | EAPC in ABCR: new regions, +3 up to 1994 and -2.14 (95% CI, -3.47 to -0.80) between 1995-97, for a total of -12.1 in 1997 vs 1989 (63.0 vs 71.6/100,000); old regions, -5.5 (-8.52 to -2.37) | RR of tumour size >2 cm, 0.74 (95% CI, 0.69-0.79); RR of N+, 0.89 (0.84-0.95); RR of Stage II+, 0.84 (0.79-0.89) | Excess RR: W aged 50-54, -6% (NS); W aged 55-59, -18% (S); W aged 64-64, -21% (S); W aged 65-69, -16% (S); total, -9% (S) | ABCR, increase from 75 to 86 in 1987-95, 98 and 96 in 1996 and 1997, fluctuation between 84 and 99 in 1998-2005 (significance, NR) |
|  | | Remarks |  | In 5 counties, the prescreening and screening epochs were not contiguous, in order to have a coverage close to zero and 100%, respectively | The implementation of the programme had a stepwise “pseudo-randomized” design for evaluation purposes |  |

**Table S1. CONTINUED**

| **Study generalities** | | | |  |  |  |
| --- | --- | --- | --- | --- | --- | --- |
|  | **First author** | | **Autier P** | **Kalager M** | Nederend J | Foca F |
|  | **Year of publication** | | **2012** | **2012** | 2012 | 2013 |
|  | **Country** | | **England** | **Norway** | The Netherlands | Italy |
|  | **Regional area(s)** | | **West Midlands** | **NA (nationwide study)** | Southern region | 700 municipalities in 6 administrative regions of central and northern Italy |
| **The screening programme** | | |  |  |  |  |
|  | **Target age (years)** | | **50-64** | **50-69** | 50-75 | 50-69 |
|  | **Target population** | | **NR** | **NR** | NR | 693,000 |
|  | **Screening interval (mos)** | | **36** | **24** | 24 | 24 |
|  | **Year of start** | | **1988** | **4 counties, 1996; the remaining 15 counties, over the following 9 years** | 1990-91 [14] | Locally varying between 1991-2005 |
|  | **Year of saturation^a^** | | **1991** | **2005** | NR | NA (see Design) |
|  | **Response rate** | | **1992-94, 70%; 1995-2004, 75%^b^** | **77%** | NR | 65% |
| **Study design and results** | | |  |  |  |  |
|  | | **Time period of observation** | **1989-2004 (no prescreening years)** | **1986-2005** | 1980-2008 | Locally varying between 1990-2006 |
|  |  | **Design** | **Joinpoint regression analysis of time trend in annual ABCR** | **Study comparing ABCR in the invited population (1998-2005, i.e. excluding the prevalence round) with the prescreening population (1987-95)** | Study of ABCR in each year 1980-2008 | Study comparing observed ABCR with expected (prescreening) ABCR, by year of screening (W aged 55-74). For each municipality, the screening years were numbered from 1 to 8 |
|  |  | **Endpoint** | **APC** | **IRR** | No numerical endpoints: curve of ABCRs as a marginal information in a study of the prevalence of advanced cancer among screened W | IRR |
|  |  | **Tumour staging** | **UICC TNM** | **UICC TNM** | UICC TNM | UICC TNM |
|  |  | **Definition of advanced stage** | **Distinct definitions: tumour size >50 mm, N+** | **Stage III+** | Stage IIA+ | T2+ |
|  |  | **Frequency of unknown stage cancer** | **NX, 20% (no time trend data)** | **NR** | Unknown stage: screen-detected cancers, 0.1% (stable); interval cancers, 0%; others and prescreening, NR | TX: year 1, 10%; year 2, 9%; thereafter, <5% |
|  | | **Results** | **>50 mm in size: APC, 0.2 (95% CI, -2.2 to 2.7) in 1989-2004. N+: increase in ABCR in 1989-92, decrease in 1993-95, stable return to the level of 1989 in 1995-2000; APC, -0.7 (-1.8 to 0.3) in 1989-2004, 1.1 (0.1-2.0) in 1992-2004** | **IRR, 0.76 (95% CI, 0.61-0.91)** | Curve interpreted as showing that ABCR was stable between 1980-2008 and did not decline after the introduction of screening | IRR: no significant changes in years 1-2, between 0.81 (95% CI, 0.75-0.88) and 0.71 (0.64-0.79) from years 3-4 onward |
|  | | **Remarks** | **ABCRs were calculated with a 33-step procedure using total incidence data (http://ci5.iarc.fr) and published tumour stage data from the screening programme [47, 48]** | **An IRR of 0.76 (95% CI, 0.61-0.91) was also observed in not-yet invited population (1996-2003 vs 1986-94)** |  | Eligibility was restricted to those municipalities in which the proportion of total incident cancers that were screen-detected (a proxy of saturation) reached the arbitrary level of 30% within year 2 |

**Table S1. CONTINUED**

| **Study generalities** | | | |  |  |  |
| --- | --- | --- | --- | --- | --- | --- |
|  | **First author** | | **Christiansen P** | **Lousdal ML** | Lousdal ML | Simbrich A |
|  | **Year of publication** | | **2014** | **2014** | 2016 | 2016 |
|  | **Country** | | **Denmark** | **Norway** | Norway | Germany |
|  | **Regional area(s)** | | **NA (nationwide study)** | **NA (nationwide study)** | NA (nationwide study) | Münster district |
| **The screening programme** | | |  |  |  |  |
|  | **Target age (years)** | | **50-69** | **50-69** | 50-69 | 50-69 |
|  | **Target population** | | **NR** | **NR** | NR | NR |
|  | **Screening interval (mos)** | | **24** | **24** | 24 | 24 |
|  | **Year of start** | | **Old regions: Copenhagen municipality, 1991; Funen county, 1993. Late regions: Bornholm municipality, 2001; West Zealand county, 2004; the remaining areas, 2007** | **One county, 1995; the remaining 18, during the following 9 years** | 1995 | 2005 |
|  | **Year of saturation^a^** | | **2010** | **2004** | 2004 | 2008 |
|  | **Response rate** | | **First screen: Copenhagen, 71%; Funen, 85%. Subsequent screens: Copenhagen, 62%; Funen, 82%** | **76% [35]** | 76% [35] | 55% |
| **Study design and results** | | |  |  |  |  |
|  | | **Time period of observation** | **1990-2011 both for early and late screening regions** | **1987-2010** | 1987-2011 | 2000-13 |
|  |  | **Design** | **Study of all-age incidence by stage, with a focus on W aged 50-69** | **Study of all-age incidence comparing ABCR in 2005-10 (screening period) vs 1987-95 (prescreening period), with a focus on W aged 50-69** | Open cohort study of ABCR in W eligible for screening vs the historic (prescreening) population of W of the same age | Log-linear Poisson regression analysis of time trend in ABCR in 2006-08 (implementation phase) and 2009-13 by 5-year age group between 45-79 |
|  |  | **Endpoint** | **ABCR** | **IRR** | IRR | Slope value from the log-linear Poisson regression model (average annual change), and absolute ABCR difference (2013 vs 2000) |
|  |  | **Tumour staging** | **UICC TNM** | **UICC TNM** | UICC TNM | UICC TNM |
|  |  | **Definition of advanced stage** | **Distinct definitions: tumour size >20 mm, N+** | **Distinct definitions: Stage II, Stage III, Stage IV** | Distinct definitions: Stage II, Stage III+ | Stage II+ |
|  |  | **Frequency of unknown stage cancer** | **NR** | **Unknown stage: 1987-95, 9%; 2005-10, 4%** | Missing information (NOS): 1987-94, 30%; 1995-2002, 19%; 2003-11, 7% | TX and/or NX, 10% (no time trend data) |
|  |  | **Results** | **>20 mm in size, transient increase in 2008-09 in old screening regions; N+, significant decline from 117 in 2001-07 to 98 in 2010-2011 in late screening regions** | **Stage II: IRR, 1.47 (95% CI, 1.40-1.55). Stage III: IRR, 1.32 (1.13-1.55). Stage IV, 0.67 (0.57-0.68). Total advanced: IRR, 1.35 (1.29-1.42)** | Stage II: IRR, 1.26 (95% CI, 1.21-1.31). Stage III+: IRR, 0.80 (0.74-0.87) | Average annual change (2009-2013): W aged 50-54, 0.016 (95% CI, -0.024 to 0.056); W aged 55-59, -0.054 (-0.095 to -0.014); W aged 60-64, -0.089 (-0.128 to -0.050); W aged 65-69, -0.113 (-0.153 to -0.073).  Absolute ABCR difference (2013 vs 2000): W aged 50-54, -0.002 (-0.191 to 0.187); W aged 55-59, -0.346 (-0.533 to -0.160); W aged 60-64, -0.279 (-0.454 to -0.105); W aged 65-69, -0.320 (-0.515 to -0.126) |
|  | **Remarks** | |  | **IRRs for W aged 20–49, who were presented as a control group for time trends in stage-specific incidence, were similar to those for W aged 50-69** | Missing stage values were multiply imputed. We report unadjusted estimates, since the purpose and necessity of adjustment were not clear. For each analysis, the IRRs for the screening vs historic group were also compared with the IRRs for the younger (ineligible) vs younger historic group. The unadjusted relative IRR was 1.14 (95% CI, 1.07-1.22) for Stage II and 1.00 (0.87-1.15) for Stage III+ | Missing stage values were multiply imputed |

**Table S1. CONTINUED**

| **Study generalities** | | | |  |  |  |
| --- | --- | --- | --- | --- | --- | --- |
|  | First author | | Autier P | Hanley JA | Jørgensen KJ | Molinié F |
|  | Year of publication | | 2017 | 2017 | 2017 | 2017 |
|  | Country | | The Netherlands | Ireland | Denmark | France |
|  | Regional area(s) | | NA (nationwide study) | 23 out of 26 counties | NA (nationwide study) | Hérault, Isère, Loire-Atlantique |
| **The screening programme** | | |  |  |  |  |
|  | Target age (years) | | 50-69 (50-75 since 1997) | 50-64 | 50-69 | 50-74 |
|  | Target population | | NR | NR | 703,289 | NR |
|  | Screening interval (mos) | | 24 | 24 | 24 | 24 |
|  | Year of start | | 1988 | 11 counties, 2000 (Region 1); 12 counties, 2007 (Region 2) | Locally varying between 1991-2007 | Locally varying during the 1990s |
|  | Year of saturation^a^ | | NR | NR | Coverage still incomplete at the end of the time period of observation | NR |
|  | Response rate | | Around 80% | 68-76% | 62- 82% | NR |
| **Study design and results** | | |  |  |  |  |
|  | | Time period of observation | 1989-2012 | 2000-13 | 1980-2010 | 2000-10 |
|  |  | Design | Multi-objective study, with a joinpoint regression analysis of time trend in annual ABCR 1989-2012 for W aged ≥50 | Multi-objective study, with a comparison of annual ABCR between Region 1 and Region2 | Multi-objective study, with a Poisson regression analysis of time trend in annual ABCR 1980-2010 | Poisson regression analysis of time trend in ABCR in 2000-10 among W aged 20-49, 50-74, and 75 and older |
|  |  | Endpoint | APC | ABCR in Region 1 minus ABCR in Region 2 as a percentage of the latter | APC, and ABCR ratio before and after the introduction of screening, both in the screening and nonscreening areas | APC |
|  |  | Tumour staging | UICC TNM | UICC TNM | UICC TNM | UICC TNM |
|  |  | Definition of advanced stage | Stage 2+ | Stage 2+ | T2+ | Distinct definitions: T2+, Stage II+ |
|  |  | Frequency of unknown stage cancer | Unknown stage: 2009-11, 1% (no time trend data) | NR | TX: 1980-2004, 8-10%; 2004-10, 4-5% | TX, 3% (no time trend data) |
|  |  | Results | APC, -0.16 (95% CI, -0.36 to 0.04) | ABCR, 20% lower in Region 1 in 2007 (significance, NR) and then narrowing | Screening areas. APC in ABCR: before screening, -0.5 (95% CI, -1.9 to 0.9); after screening, -1.1 (-1.8 to -0.3). ABCR ratio, 0.96 (0.90 to 1.02) | W aged 50-74. T2+: APC, - 1.9 (95% CI, -2.8 to -1.0). Stage II+: APC, -2.0 (-2.7 to -1.3) |
|  | | Remarks | Stage 2+ probably indicates T2+, since a tumour size of 20 mm is referred to as the threshold size to distinguish between Stage 1 and 2 | It is not clear whether Stage 2+ indicates Stage II+ | Nonscreening areas. APC in ABCR: before screening, 1.7% (95% CI, 0.8% to 2.6%); after screening, 3.0% (2.6% to 3.3%). ABCR ratio, 1.46 (1.41 to 1.52) | Overall, a 20.9% linear decrease in T2+ cancer over 11 years in the three screening areas were noted for W aged 50-74. No change in ABCR was found in younger or older W |

**Table S1. CONTINUED**

| **Study generalities** | | |  |
| --- | --- | --- | --- |
|  | First author | Puliti D | Larsen IK |
|  | Year of publication | 2017 | 2018 |
|  | Country | Italy | Norway |
|  | Regional area(s) | Nine health care districts in central and northern Italy | NA (nationwide study) |
| **The screening programme** | |  |  |
|  | Target age (years) | 50-69 | 50-69 |
|  | Target population | 413,000 | NR |
|  | Screening interval (mos) | 24 | 24 |
|  | Year of start | Locally varying between 1991-98 | 1996 |
|  | Year of saturation^a^ | Locally varying between 1993-2000 | 2005 |
|  | Response rate | NR | 75% |
| **Study design and results** | |  |  |
|  | Time period of observation | Locally varying between 1991-2011 | 1980-2015 |
|  | Design | Cohort study of ABCR in attenders to screening vs non-attenders, with a comparison of the observed number of ABC among W invited with that expected based on prescreening ABCR | Study of all-age stage-specific incidence based on different staging systems, with a focus on W aged 50-69 |
|  | Endpoint | O:E ratio | No numerical endpoints: curve of ABCRs |
|  | Tumour staging | UICC TNM | UICC TNM |
|  | Definition of advanced stage | T2+ | Distinct definitions:  Stage II, Stage III, Stage IV |
|  | Frequency of unknown stage cancer | TX, 10-29% (no time trend data) | Unknown stage, 40% with an apparent increase in the first half of the time period of observation and an apparent decrease in the second |
|  | Results | T2+: O:E ratio, 0.83 (95% CI, 0.80-0.86) | Curves interpreted as showing an incidence decrease for Stage II and an increase for Stage III |
|  | Remarks | The O:E ABC ratio was estimated for the purposes of assessment of self-selection bias |  |

^a^ Year of saturation: the year by which all women in the initial target population were invited at least once.

^b^ Indirectly derived or calculated from numbers, tables, and figures in the paper.
